# Cerebral cortex dose sparing for glioblastoma patients: IMRT versus robust treatment planning

**DOI:** 10.1186/s13014-018-0953-x

**Published:** 2018-02-06

**Authors:** Ann-Katrin Exeli, Daniel Kellner, Lukas Exeli, Phil Steininger, Frank Wolf, Felix Sedlmayer, Heinz Deutschmann

**Affiliations:** 1 Department of Radiotherapy and Radio-Oncology at the University Hospital Salzburg, Landeskrankenhaus, Paracelsus Medical University, 5020 Salzburg, Austria; 2 Institute for Research and Development on Advanced Radiation Technologies (radART), Paracelsus Medical University, 5020 Salzburg, Austria

**Keywords:** Radiotherapy, Robust treatment planning, IMRT, Glioblastoma, Cortex, Cortical sparing

## Abstract

**Background:**

To date, patients with glioblastoma still have a bad median overall survival rate despite radiation dose-escalation and combined modality treatment. Neurocognitive decline is a crucial adverse event which may be linked to high doses to the cortex. In a planning study, we investigated the impact of dose constraints to the cerebral cortex and its relation to the organs at risk for glioblastoma patients.

**Methods:**

Cortical sparing was implemented into the optimization process for two planning approaches: classical intensity-modulated radiotherapy (IMRT) and robust treatment planning. The plans with and without objectives for cortex sparing where compared based on dose-volume histograms (DVH) data of the main organs at risk. Additionally the cortex volume above a critical threshold of 28.6 Gy was elaborated. Furthermore, IMRT plans were compared with robust treatment plans regarding potential cortex sparing.

**Results:**

Cortical dose constraints result in a statistically significant reduced cerebral cortex volume above 28.6 Gy without negative effects to the surrounding organs at risk independently of the optimization technique. For IMRT we found a mean volume reduction of doses beyond the threshold of 19%, and 16% for robust treatment planning, respectively. Robust plans delivered sharper dose gradients around the target volume in an order of 3 – 6%. Aside from that the integration of cortical sparing into the optimization process has the potential to reduce the dose around the target volume (4 – 8%).

**Conclusions:**

We were able to show that dose to the cerebral cortex can be significantly reduced both with robust treatment planning and IMRT while maintaining clinically adequate target coverage and without corrupting any organ at risk. Robust treatment plans delivered more conformal plans compared to IMRT and were superior in regards to cortical sparing.

## Background

Glioblastoma multiforme (GBM) is a highly malignant brain tumor with poor prognosis. With surgical resection alone the median survival amounts 6 months [1]. Today the gold standard treatment is based on a multidisciplinary approach employing surgery with maximal safe resection followed by radiotherapy with daily concomitant temozolomide [2] followed by additional cycles of temozolomide. The overall survival with the combined modality treatment is in the range of 27% at 2 years and 10% at 5 years [3]. With the growing number of long term survivors it is essential to assure that the post therapeutic life time is not compromised by cognitive dysfunction and its negative impact on social and professional functioning and self-care. Meyers et al. reported that 50 – 90% of patients who survived more than 6 months after fractionated brain irradiation suffered from radiation-induced cognitive impairment [4]. Cognitive deficits are without limitation linked to radiation-induced tissue damage. The pathomechanism is not clearly understood and to date there is no effective prevention [5]. It may develop from vascular injury, oxidative stress, radionecrosis and cortical atrophy. Karunamuni et al. described cerebral cortical atrophy with greater extent at high dose regions. They found a critical dose threshold of 28.6 Gy to result in a 20% probability of severe atrophy [6, 7].

Typically, a margin around the clinical tumor volume (CTV) compensates for the uncertainties which may occur during the treatment process in radiotherapy – this method is called planning target volume (PTV)-concept. Unfortunately, the approach to achieve good tumor control via rigid margins may come at the price of increased dose to the surrounding tissue. A new concept for uncertainty management is called robust treatment planning. Robust treatment planning does not use margins around the CTV [8, 9]. It accounts for possible uncertainties during the optimization process, which can lead to sharper dose gradients and better sparing of organs at risk (OARs) due to a higher flexibility for the optimizer compared to the margin-based approach. The idea to incorporate uncertainty analysis and robustness directly into the treatment planning process is over 30 years old [10], however, it is only available since a few years in commercial treatment planning systems. Early papers concerning robust optimization focused on its methodology with the demonstration of extreme cases like spinal cord tumors. Current research investigates when and how to use robust optimization for common tumor sites. In pancreatic cancer, the dosimetric benefit was limited [11]. Fontanarosa described a reduction of dose to OARs for robust optimization compared to the traditional margin-based IMRT plans for head and neck cancer patients [12]. In the treatment of prostate cancer, robust treatment planning could also be beneficial [13]. For oropharyngeal cancer patients, robustness recipes for intensity modulated proton therapy were published [14]. Moreover, new treatment techniques like volumetric arc therapy (VMAT) with simultaneous integrated boost (SIB) or proton radiation therapy seem to be promising for further dose sparing in crucial neuronal structures [15–17].

In the treatment of glioblastomas, the sensitive cerebral cortex is often located close to the tumor, thus we hypothesized robust treatment plans might deliver better plans than IMRT. We evaluated cerebral cortical dose avoidance in inverse treatment plan optimization for IMRT and robust treatment plans in comparison to both: the dosimetric cost to the target volume and ambient organs at risk.

## Methods

### Patients

The cohort comprised ten patients diagnosed with glioblastoma multiforme (7 female, 3 male) with a median age of 62.5 years (range 39 – 66 years) who were irradiated between 2013 and 2014 at our department. Six patients received adjuvant chemoradiation, five of which had residual disease. Four patients received primary chemoradiation.

### Treatment planning preparation

The initial treatment planning computed tomography (CT)-images were acquired with a *Somatom Emotion [Siemens]* and a slice thickness of 2.5 mm. CT-images were fused with contrast-enhanced T1-weigthed magnetic resonance imaging (MRI, *Philips*) sequences. To reduce interobserver variability only one radiooncologist expertised in central nervous system (CNS) treatment contoured the target volumes as well as organs at risk for all patients following the ESTRO-ACROP (*European Society for Radiotherapy and Oncology – Advisory Committee on Radiation Oncology Practice*) guidelines for target delineation of glioblastomas [18, 19]. The dose limits for OARs are depicted in Table 1. For cochlea, lens and brain stem reported dose constraints are inconsistent thus the lower primary objective may be disregarded depending on the specific clinical situation. If the ipsilateral optic nerve was in close proximity to the PTV, target coverage was prioritized.

**Table 1:** OARs dose limits in glioblastoma patients with primary and secondary objectives

Organs at risk	Objective(s)	References
Brainstem	D_max_ < 54 Gy	[32, 33]
	1-10 cm^3^ < 59 Gy	[33]
Cochlea	D_mean_ ≤ 45 Gy	[34, 35]
	D_mean_ < 50 Gy	[32]
Cortex	D_max_ < 28.6 Gy	[6]
Eyes	D_max_ ≤ 45 Gy	[36]
Lens	D_max_ < 6 Gy	[18]
	D_max_ < 10 Gy	[32, 37]
Optic nerves	D_max_ < 54 Gy	[29, 32]

### Re-planning with IMRT and robust optimization

Planning was performed for the whole patient collective based on CT-imaging. Dose was prescribed to 60 Gy delivered in 30 fractions using the dose objectives summarized in Table 1, except for plans without cortex optimization where the cortex objective was not applied. All plans were calculated in the treatment planning system *RayStation* 4.7 *[RaySearch Laboratories],* which uses minimax optimization for the robust approach [9]. The treatment plans consisted of 13 equidistant beams with 6 MV photon energy starting at a gantry angle of 0°. The PTV was defined as CTV plus an isotropic margin of 3 mm which is routinely used in our department. Initially, step-and-shoot IMRT plans were generated with 95% dose coverage of the PTV at 100% prescription dose. Subsequently robust treatment plan optimization was performed on the same patient analog to the IMRT- *International Commission on Radiation Units and Measurements* (ICRU) conform target isodose coverage of −5% to +7%, however to the CTV instead of the PTV [20]. All other optimization settings were set identically for IMRT and robust optimization including the values of the setup error which was set to 3 mm for robust planning corresponding to the CTV-PTV margin used for IMRT planning.

To prevent optimization conflicts cortex structures were cropped if they overlapped with a technical PTV(t), which had been created by further expansion of the PTV by 10 mm in order to create a minimum distance of 10 mm between PTV and cortex.

To assess the gradient sharpness of the dose fall off around the PTV additional ringstructures were generated to evaluate the average dose 10 and 20 mm outside of the PTV (PTV + 10, PTV + 20) as well as to the surrounding cortex in a distance of 20 mm from the PTV (CortexPTV + 20).

We investigated two questions that go hand in hand: Does the benefit of cortex sparing outweigh the increased workload for contouring and if so, is robust treatment planning preferable to IMRT?

## Results

The median CTV volume was 133 cm^3^ (range 27 – 292 cm^3^). The tumor locations were: left (7), right (2), bilateral (1) and temporal (4), parietal (4), occipital (1) and frontal (1). In one patient, accurate MR image fusion was not possible. Therefore he was excluded from the analysis for cortex sparing.

### Integration of cortex sparing

**Fig. 1:**
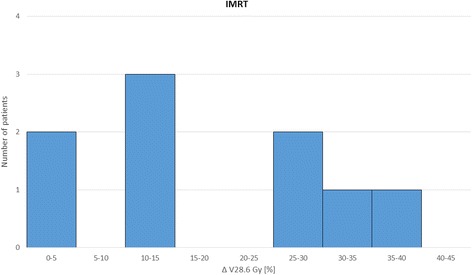
Difference of the volume above 28.6 Gy in percent (Δ V28.6 Gy) with and without cortex sparing for IMRT plans

**Fig. 2:**
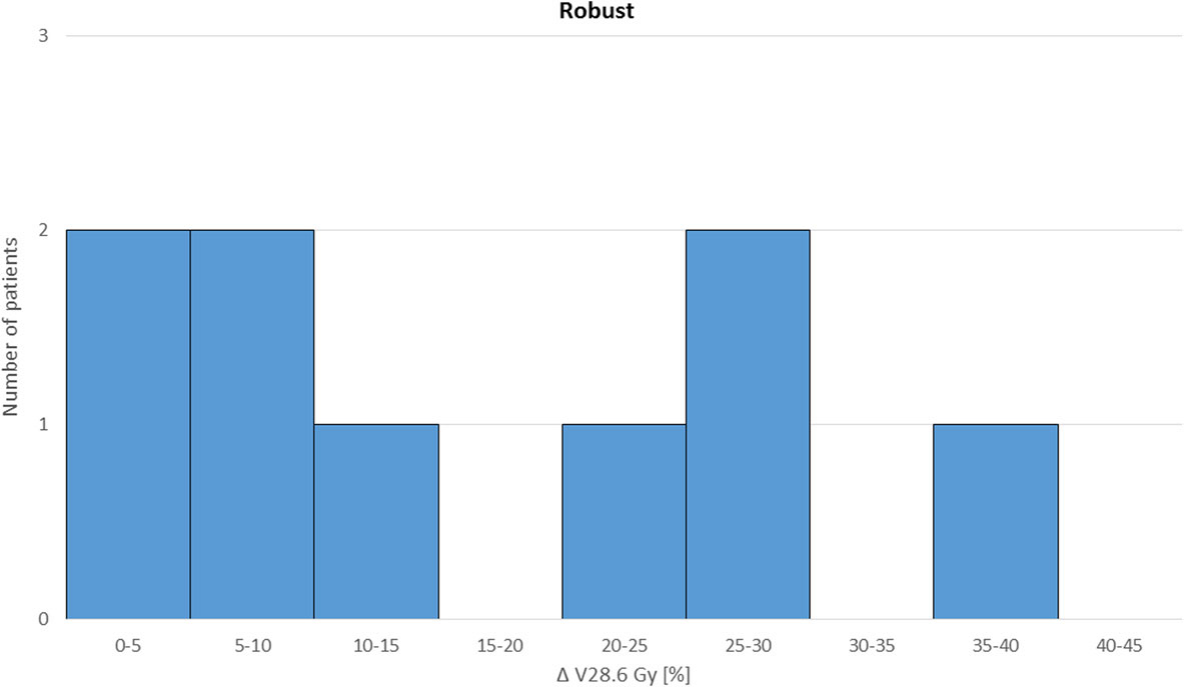
Difference of the volume above 28.6 Gy in percent (Δ V28.6 Gy) with and without cortex sparing for robust plans

Figures 1 and 2 demonstrate the potential of the integration of cortical sparing into the optimization process. The difference of the average volume of both cortices receiving more than 28.6 Gy (ΔV28.6 Gy [%]) is illustrated in percent for the whole patient cohort for IMRT (Fig. 1) and for robust optimization (Fig.2).

For both the ipsi- and contralateral cortex the mean volume above the threshold of 28.6 Gy was significantly reduced (*p* < 0.01) by the implementation of cortex sparing in both IMRT and robust treatment planning. Resulting in a *p*-value for the ipsi- and contralateral cortex of 0.0034/0.006 (IMRT) and 0.0052/0.0093 (robust). As hypothesized the dose to the ipsilateral cortex was lower with robust treatment planning compared to IMRT, independently of the cortex optimization (*p*-value 0.0021 without CS, 0.0059 with CS). There was no statistical significant difference between IMRT and robust treatment planning for the contralateral cortex (*p*-value >0,01).

The average near maximum dose (D1) and the average median dose (D50) to the OARs over the complete patient cohort with respect to the different treatment techniques, with and without cerebral cortex objectives, is recapitulated in Table 2. The values are given in Gy plus minus one standard deviation. The integration of cortex sparing into the optimization process had no negative influence on the doses to other OARs or the target coverage independent of the planning approach: robust or IMRT. By integrating cortex objectives of 28.6 Gy we found an average volume reduction of 16% to 19% for every patient (range: 2 – 42%). Also the near maximum dose to the cortex was markedly reduced with cortex objectives in the range of 8% to 14%.

**Table 2:** Dose to the OARs for different treatment techniques

OAR	IMRT	IMRT with CS	Robust	Robust with CS
TV^a^ (D50)	60.0	60.0	60.0	60.0
	± 0.0	± 0.0	± 0.0	± 0.0
Brainstem (D1)	51.8	51.7	51.3	50.8
	± 17.5	± 17.5	± 17.5	± 17.3
Cerebellum (D50)	15.5	15.2	14.3	14.3
	± 17.1	± 17.4	± 17.3	± 17.5
Cortex ipsi^b^ (D1)	48.9	35.0	43.8	33.6
	± 3.9	± 3.3	± 4.6	± 3.0
Cortex contra^b^ (D1)	44.1	33.7	41.0	32.6
	± 10.4	± 5.8	± 10.0	± 5.7
Hemisphere ipsi (D50)	37.4	32.5	34.2	30.7
	± 23.1	± 20.5	± 22.8	± 20.3
Hemisphere contra (D50)	21.1	17.5	19.4	16.5
	± 13.1	± 10.2	± 12.9	± 10.4
Eye ipsi (D1)	12.6	11.7	13.3	12.3
	± 7.4	± 6.8	± 8.6	± 7.3
Eye contra (D1)	11.2	10.3	10.3	10.3
	± 6.9	± 6.1	± 6.3	± 6.4
Lens ipsi (D1)	4.1	4.1	4.2	4.3
	± 2.0	± 2.2	± 2.2	± 2.2
Lens contra (D1)	3.7	3.6	3.5	3.4
	± 1.9	± 2.0	± 2.0	± 2.0
Optic nerve ipsi (D1)	36.1	33.5	34.8	31.7
	± 21.0	± 19.9	± 20.1	± 18.8
Optic nerve contra (D1)	27.1	23.4	25.8	22.2
	± 15.5	± 13.7	± 15.7	± 13.3
Cochlea ipsi (D50)	23.6	23.2	22.6	22.0
	± 20.9	± 20.8	± 21.3	± 21.1
Cochlea contra (D50)	9.2	8.7	8.4	7.9
	± 9.8	± 9.5	± 9.5	± 9.2
Hippocampus ipsi (D50)	43.0	41.4	41.5	40.4
	± 22.3	± 22.6	± 22.5	± 22.5
Hippocampus contra (D50)	30.7	27.0	29.2	25.8
	± 12.8	± 10.7	± 12.4	± 10.3

Comparison of average median dose (D50) or the average near maximum dose (D1) in Gy plus minus one standard deviation to the target volume (TV) and OARs in robust and IMRT plans with and without cortex sparing. Some OARs are divided into ipsilateral (ispi) and contralateral (contra) hemisphere

^a^TV: CTV for robust optimization, PTV for IMRT

^b^Cortex complies the technical OAR; *CS* Cortex sparing

**Fig. 3:**
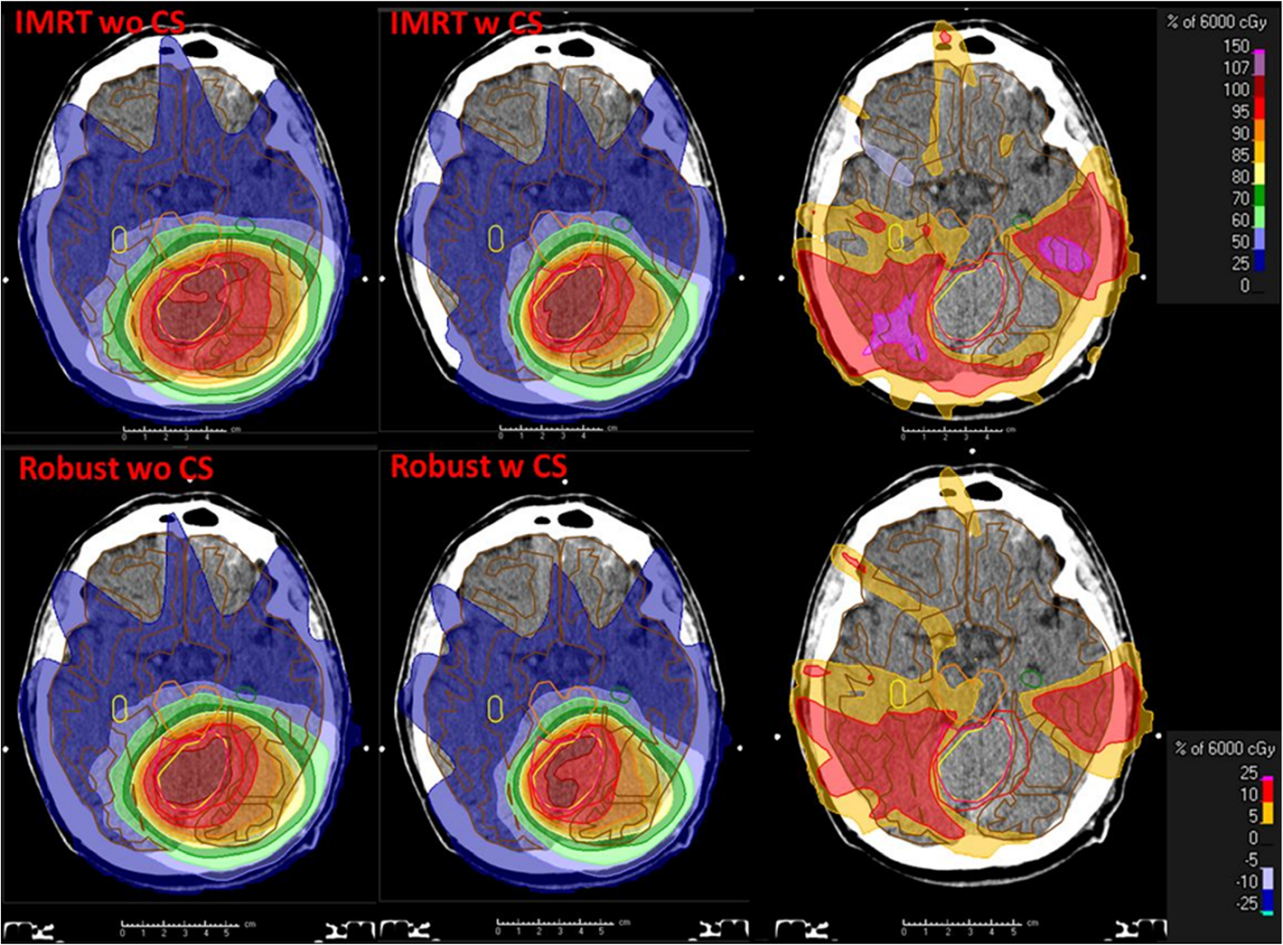
IMRT and robust dose distributions for patient A on a representative CT slice. Left column: IMRT/ robust plan without cortex sparing (wo CS). Middle column: IMRT/ robust plan with cortex sparing (w CS). Right column: dose difference (plan wo CS minus plan w CS). The dose difference shows the potential of cortical sparing of up to 25% (purple area) for this slice. Red and yellow areas indicate a dose reduction of 10%, and 5%, respectively

**Fig. 4:**
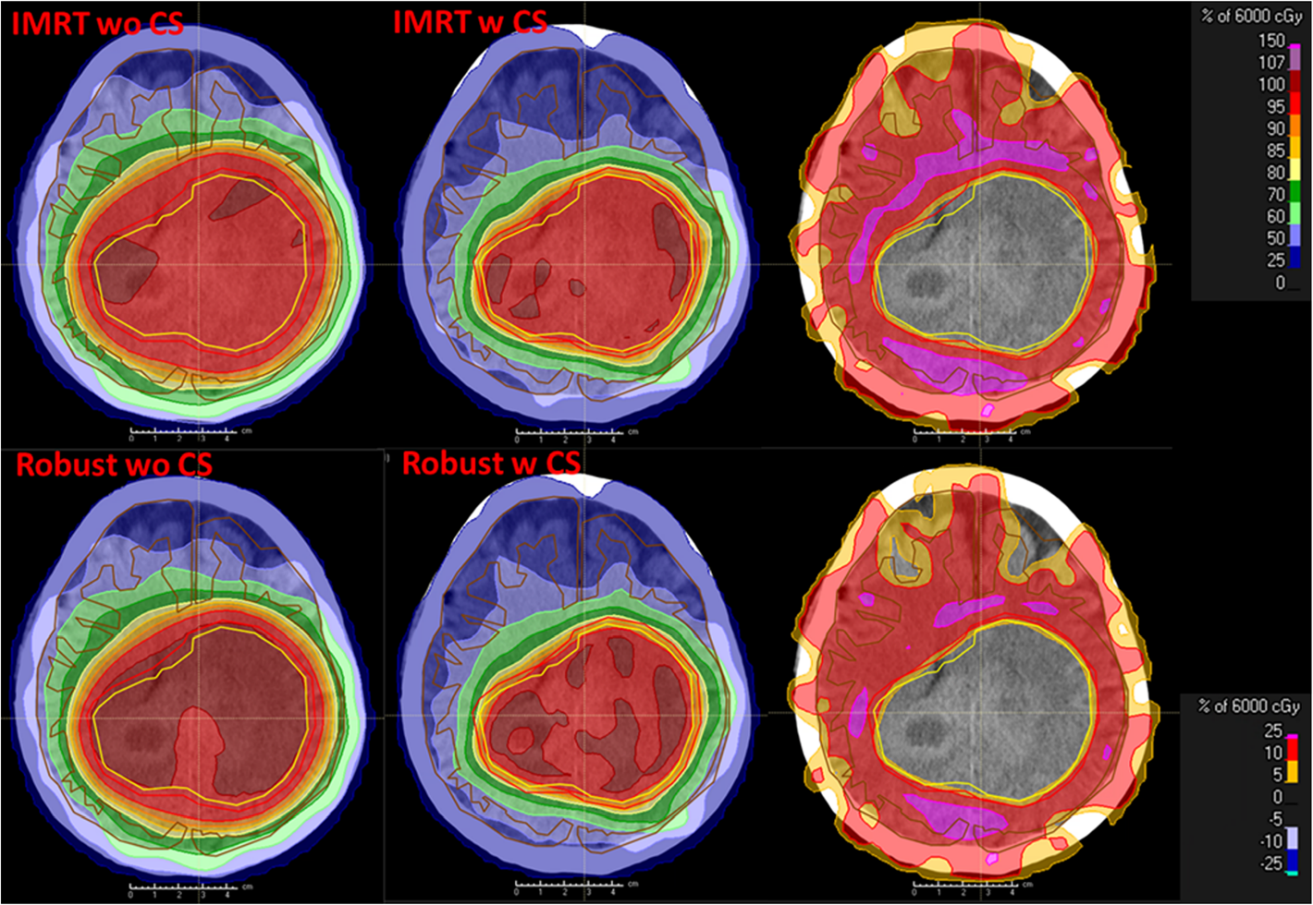
IMRT and robust dose distributions for patient B on a representative CT slice. Left column: IMRT/ robust plan without cortex sparing (wo CS). Middle column: IMRT/ robust plan with cortex sparing (w CS). Right column: dose difference (plan wo CS minus plan w CS). The dose difference shows the potential of cortical sparing of up to 25% (purple area) for this slice. Red and yellow areas indicate a dose reduction of 10%, and 5%, respectively

In Figs. 3 and 4 the impact of cortex sparing on the mean doses of organs at risk is shown. The dose distributions without (wo, left) and with (w, middle) cortical sparing for IMRT and robust planning is illustrated on a representative CT slice for two patients with different tumor size and location. The dose difference (right) shows the potential of cortical sparing of up to 25% for the representative slice. Red and yellow areas indicate a dose reduction of 10%, and 5%, respectively.

### Dose gradient around the PTV

To evaluate the sharpness of the dose gradient around the PTV we generated two structures: The PTV + 10 and the PTV + 20, which are defined as ringstructures outside the PTV with a radius of 10 mm and 20 mm respectively. The structure CortexPTV + 20 considered only the cortex within 20 mm to the PTV. The integration of cortex sparing into the optimization process had the potential to reduce the average dose around the PTV in a magnitude of 4 – 8%, depending on the treatment technique and the distance from the PTV. The smallest effect was seen for robust treatment planning in the PTV + 10 and the largest effect for IMRT in the PTV + 20. The cortex in close proximity to the PTV (CortexPTV + 20), which is defined as the intersection between the cortex and the structure PTV + 20, was spared around 16 – 19% using cortex objectives (Table 3).

**Table 3:** Dose around the PTV

	Average Dose in Gy		
	PTV + 10	PTV + 20	CortexPTV + 20
IMRT	56.1 ± 0.9	44.9 ± 3.5	41.4 ± 4.9
IMRT with CS	53.5 ± 0.7	41.3 ± 2.8	33.4 ± 3.6
Robust	53.9 ± 1.5	42.1 ± 3.9	37.2 ± 4.9
Robust with CS	51.7 ± 0.9	39.4 ± 3.1	31.3 ± 3.6

Average dose for the patient cohort around the PTV within a distance of 10 mm (PTV + 10) and 20 mm (PTV + 20) as surrogate for the dose gradient in Gy plus minus one standard deviation. For the evaluation of cortex sparing the values of CortexPTV + 20 indicate the average dose to the cortex in a 20 mm distance from the PTV for IMRT and robust plans with and without cortex sparing (CS)

## Discussion

The risk of occurrence and the severity of radiation-induced cognitive impairment seems to be correlated with the dose to and the magnitude of the irradiated volume of several critical structures for neurocognition. Thus, in whole brain radiotherapy, attention has been paid to spare uninvolved tissues deemed to be capable of neuroregeneration. Cortical damage seems to have an impact on neurocognitive deficits. Quite recently, the important role of the cortex for cognitive processes was emphasized. Eichenbaum et al. stated that the prefrontal cortex may play an equivalent role in the organization of memories as the hippocampus [21]. For instance, recommendations for hippocampal sparing during brain irradiation have been elaborated for several years. Hippocampal sparing is currently evaluated in a multicenter study, the results of which are expected to be presented in 2019 [22]. The hippocampus has been shown to be important for several neurocognitive functions [23–26]. Thus, additionally to the common known structures like hippocampus, subventricular zone, amygdale and thalamus, it may be essential to focus also on the cerebral cortex as an important OAR [27, 28].

We have investigated two different planning strategies, regular IMRT implementing a CTV-PTV margin concept and robust planning to the CTV and tested them for their potential to spare the cortex as well as other critical OAR’s in brain irradiation.

We were able to validate a clear benefit for the implementation of cortex sparing into the treatment planning optimization process. This advantage was seen for both optimization techniques – IMRT and robust – with a significant superiority of robust plans. As hypothesized even without cortical optimization robust plans resulted in significantly less V28.6 Gy to the ipsilateral cortex. This is supported by the results of the evaluation of the sharpness of the dose gradient (Table 3) where the robust plans delivered a better conformity than the IMRT plans, most likely due to the prescription to the CTV rather than the PTV plus the additional information in the robust optimization process compared to the rigid margin concept, which enables more conformal plans plus the additional flexibility in the robust optimization process compared to the rigid margin concept. We claim that this added flexibility helps to strike a better dose reduction for surrounding healthy tissue, in our case the cerebral cortex.

The benefit of cortex sparing was apparent for every patient. The magnitude of the benefit depended primarily on the size of the target volume and its proximity and spatial extent to the cerebral cortex. Patients with large CTV volumes (> 200 ml) appeared to have the greatest benefit using cortex optimization, whereas for patients with small CTV volumes located centrally in the brain hemisphere it did not seem to be as crucial to integrate cortical sparing into the optimization process. The residual organs at risk, in particular hippocampus, brainstem, cerebellum, brain hemispheres, eyes, lenses, optic nerves and cochleae, had no detriment when cortical sparing was implemented in the optimization process (Table 2). However, dose avoidance to the cerebral cortex seemed to be associated with a small increase in heterogeneity of the target volume coverage, which has also been described by Karunamuni [6].

A limitation of our study is the small sample size resulting in high standard deviations with regard to dose-volume distributions for OARs, especially the V28.6 Gy for the cortex. However, we believe that by choosing a heterogenous patient collective with glioblastomas of different sizes and locations our results are universally applicable and representative for most glioblastoma cases.

In terms of dose sparing to critical organs at risk proton therapy has been shown to be superior to standard photon therapy [29, 30] due to its physical properties and the used scanning beam technology. However, because of the cost and limited availability of proton centres standard photon radiotherapy will continue to play a major role in the treatment of glioblastomas warranting the continued effort to optimize its efficiency. Chatterjee et al. published a study with a knowledge-based radiation photon therapy model for GBM patients where they reduced the planning time down to 7 min for IMRT, compared to a typical 4 h for manual planning [31].

To the best of our knowledge, this is the first publication of robust optimization for radiotherapy of glioblastoma patients. We present a first recommendation to prefer robust optimization with a cortex sparing approach.

## Conclusion

By implementation of cortex sparing the mean and maximum dose to the cerebral cortex can be significantly reduced both with IMRT and robust treatment planning, while maintaining clinically adequate target coverage and without corrupting any organ at risk. Despite the high standard deviations, a significant volume reduction above the critical threshold of 28.6 Gy for the cortex could be shown independent of treatment technique and cortex side. Robust treatment planning was shown to be superior to IMRT with regards to the ipsilateral cortex and the dose fall off around the target volume.

Therefore we recommend integration of cortex sparing into the treatment plan optimization process for glioblastoma patients in particular when the target volume abuts on larger parts of the cortex.

## Abbreviations

CNS: Central nervous system
CS: Cortex sparing
CT: Computed tomography
CTV: Clinical target volume
DVH: Dose-volume histograms
ESTRO-ACROP: European Society for Radiotherapy and Oncology – Advisory Committee on Radiation Oncology Practice
GBM: Glioblastoma multiforme
ICRU: International Commission on Radiation Units and Measurements
IMRT: Intensity-modulated radiotherapy
MRI: Magnetic resonance imaging
OARs: Organs at risk
PTV: Planning target volume
TV: Target volume
